# Human-induced pluripotent stem cell-derived macrophages and their immunological function in response to tuberculosis infection

**DOI:** 10.1186/s13287-018-0800-x

**Published:** 2018-02-26

**Authors:** Danping Hong, Jiongyan Ding, Ouyang Li, Quan He, Minxia Ke, Mengyi Zhu, Lili Liu, Wen-Bin Ou, Yulong He, Yuehong Wu

**Affiliations:** 1 College of Life Science, Zhejiang Sci-tech University, 928 Second Avenue, Xiasha Higher Education Zone, Hangzhou, China; 2 Zhejiang Provincial Key Laboratory of Silkworm Bioreactor and Biomedicine, Hangzhou, 310018 China

**Keywords:** Human induced pluripotent stem cells, THP-1, Macrophages, BCG

## Abstract

**Background:**

Induced pluripotent stem cells (iPS) represent an innovative source for the standardized *in vitro* generation of macrophages (Mφ). Mφ show great promise in disease pathogenesis, particularly tuberculosis. However, there is no information about human iPS-derived (hiPS) macrophages (hiPS-Mφ) in response to tuberculosis infection.

**Methods:**

In the present study, macrophages derived from hiPS were established via embryoid body (EB) formation by using feeder-free culture conditions, and the human monocyte cell line THP-1 (THP-1-Mφ) was used as control. iPS-Mφ were characterized by using morphology, Giemsa staining, nonspecific esterase staining (α-NAE), phagocytosis, and surface phenotype. Additionally, after treatment with Bacillus Calmette-Guérin (BCG) for 24 h, cell apoptosis was detected by using an Annexin V-FITC Apoptosis Detection assay. The production of nitric oxide (NO), expression of tumor necrosis factor alpha (TNF-α), activity of apoptosis-related protein cysteine-3 (Caspase-3) and expression of B-cell lymphoma-2 (Bcl-2) were analyzed.

**Results:**

With respect to morphology, surface phenotype, and function, the iPS-Mφ closely resembled their counterparts generated *in vitro* from a human monocyte cell line. iPS-Mφ exhibited the typically morphological characteristics of macrophages, such as round, oval, fusiform and irregular characteristics. The cells were Giemsa-stained-positive, α-NAE-positive, and possessed phagocytic ability. iPS-Mφ express high levels of CD14, CD11b, CD40, CD68, and major histocompatibility complex II (MHC-II). Moreover, with regard to the apoptotic rate, the production of NO, expression of TNF-α, and activity of Caspase-3 and Bcl-2, iPS-Mφ closely resemble that of their counterparts generated *in vitro* from human monocyte cell line in response to BCG infection. The rate of apoptosis of BCG-treated iPS-Mφ was 37.77 ± 7.94% compared to that of the untreated group at 4.97 ± 1.60% (*P* < 0.01) by using Annexin V-FITC Apoptosis Detection. Additionally, the rate of apoptosis of BCG-treated THP-1-Mφ was 37.1 ± 2.84% compared to that of the untreated group at 6.19 ± 1.68% (*P* < 0.001). The expression of TNF-α and the production of NO were significantly increased (*P* < 0.001), and the activity of Caspase-3 was increased. However, the expression of Bcl-2 was inhibited (*P* < 0.001).

**Conclusions:**

Our results demonstrate that Mφ derived from hiPS perform the immunological function in response to Bacillus Calmette-Guérin infection by undergoing apoptosis, increasing the production of NO and expression of TNF-α. Thus, our study may help to overcome the limitations of research into certain rare diseases due to the lack of adequate supply of disease-specific primary cells.

**Electronic supplementary material:**

The online version of this article (10.1186/s13287-018-0800-x) contains supplementary material, which is available to authorized users.

## Background

Macrophages (Mφ) are one of the most important immune cells in the body and are distributed in most tissues and organs. These cells play a central role in the non-specific immune clearance of bacteria, viruses, fungal pathogens, and specific immune response antigen presentation and corresponding cytokine production [1]. While traditionally tissue-resident, Mφ have been regarded as being continuously replenished from hematopoietic stem cells via the intermediate step of peripheral blood monocytes, this concept has recently been challenged [2].Thus, studies have recently demonstrated that substantial populations of Mφ are prenatally seeded and exhibit considerable longevity (months to years); in addition, these cells have, at least in part, self-renewal potential [3, 4].

Additionally, CD34+ hematopoietic stem cells, monocytes and early T lymphocytes, etc. [5] can be differentiated into macrophages under certain conditions. There are two main sources of human macrophages in vitro. One source is the cell lines derived from tumors, such as U937 and THP-1, and the other source is primary cells, such as peripheral blood mononuclear cells. The macrophages derived from the former have the potential for unlimited replication and play an important role in macrophage-related biology research [6, 7]. However, compared with primary macrophages, these immortalized cell lines are prone to abnormal genetic structure changes, leading to a lack of function, severely limiting their application in related research [6, 7]. Macrophages derived from peripheral blood represent macrophages that cannot self-renew, and each study requires large amounts of blood from the donor and depends on the donor’s physiological state and genes, etc., resulting in test results that are not representative.

Following the groundbreaking report by Takahashi and Yamanaka in 2006 [8], induced pluripotent stem cells (iPS) provide a new method and pathway for the screening of drugs and individual-specific treatments. Studies have demonstrated that humans, mice, pigs, sheep, guinea pigs, rhesus monkeys, and marmosets [9–13], and other species of various somatic cells, such as fibroblasts, peripheral blood cells, and amniotic fluid cells [14, 15], can be reprogrammed into iPS without the limitation of the donor age [16]. Now, human iPS (hiPS) can easily be generated by a number of reprogramming techniques and from various cell and tissue sources. Compared with traditional stem cell technology used in research, iPS technology not only avoids the ethical and moral limitations of stem cell research, but could also create an inexhaustible source of cells that could be used to derive the differentiated cells required for patient-specific therapy, such as macrophages, cardiomyocytes, neurons, and pancreatic beta cells [17, 18]. However, successful iPS-based gene and cell therapy relies on the efficient differentiation of iPS into the desired cell types. Whereas substantial progress has been made to differentiate hiPS into defined mature hematopoietic cells, such as granulocytes, macrophages, erythrocytes, megakaryocytes or dendritic cells, lymphoid differentiation or the generation of long-term repopulating hematopoietic stem cells from humans remain hampered by the low quality and inefficient quantity of the output cells [2].

Studies have shown that iPS can be differentiated into immune cells under appropriate induction conditions [19–21]. The macrophages in immune cells are the most plastidic cells in the hematopoietic system and exist in all tissues and play many different roles in development [22], in vivo balance, tissue repair, immune response to pathogens [23, 24], and primary ovarian insufficiency [25]. Thus, the use of iPS-derived macrophages to establish a related disease model can further analyze the relationship between macrophages and disease. Although, many methods such as direct differentiation by the addition of different factors [26], the embryoid body (EB) formation method [27, 28], and co-culture with bone marrow stromal cells (OP9) [17] have been used for the differentiation of iPS into macrophages, the efficiency remains low, and the differentiation system remains unstable.

Furthermore, the generation of hiPS-derived macrophages focuses on the application of therapy or the pathogenesis of cancer [29, 30], Mendelian disease [31], Alzheimer’s disease [30], HIV [32], *Chlamydia trachomatis* infections [33], chronic granulomatous disease [34], and X-linked chronic granulomatous disease [35]. Unfortunately, many questions about the mechanisms of hiPS-derived macrophages in disease pathogenesis remain. Furthermore, macrophages show great promise in disease pathogenesis, particularly tuberculosis. Tuberculosis is a zoonotic infectious disease and a serious threat to human health. As the main host cells to invasive *Mycobacterium tuberculosis* (MTB), macrophages interact with MTB, playing a crucial role in the occurrence and development of tuberculosis. Studies of these interactions have confirmed a crucial role for these cells in the occurrence and development of tuberculosis. However, there is no information about hiPS-derived macrophages in response to tuberculosis infection. In particular, their effects on tuberculosis infection, especially the immunological function in response to tuberculosis infection, have not been thoroughly investigated.

Thus, in the present study, we optimized the method used to generate these cells by using an EB-forming method combined with the addition of different factors to differentiate iPS into monocytes and subsequently mononuclear cells into macrophages. These investigations led to development of a stable experimental culture condition for human iPS differentiation. Using Western blot analysis, immunostaining and through a combination of flow cytometric analyses, we elucidated the immunological function of hiPS-derived macrophages (iPS-Mφ) in response to Bacillus Calmette-Guérinin (BCG) a similar manner to Mφ derived from human monocyte cells.

## Methods

### Cell culture

Human iPS (DYR0100 cells) and human embryonic stem (ES) (ZQ0271) were cultured under feeder-free culture conditions in chemically defined mTeSR1 medium (Stemcell Technologies, Vancouver, BC, Canada) on Matrigel (Corning, Corning, NY, USA)-coated dishes.

Human monocyte cells (THP-1) were cultured in RPMI 1640 medium supplemented with 10% fetal bovine serum (FBS, Gibco, Waltham, MA, USA), 0.1 mg/ml penicillin and 0.05 mg/ml gentamicin.

### Mφ differentiation of iPS

iPS-derived Mφ and ES-derived Mφ were generated using a modified version of a previously established protocol [31, 36–39]. The hiPS were gently digested with Accutase (Stemcell Technologies), and 2 × 10^6^ cells were resuspended in Knockout-DMEM medium (KO-DMEM, Gibco) supplemented with 10% Knockout-serum replacement (KSR, Gibco), 1% Non-essential amino acids (NEAA, Gibco), 1 mM L-Glutamine (Sigma-Aldrich,, St. Louis, MO, USA), 50 μM β-mercaptoethanol (β-ME, Solarbio, Beijing. China) and 10 μM ROCK-inhibitor (Y-27632, Selleckchem, Houston, TX, USA) and cultured on 6-well ultralow attachment plates (Corning) for 24 h. The medium was changed daily. After 8–11 days, EB were seeded onto gelatin-coated 24-well plates in DMEM (Gibco) medium supplemented with 10% FBS, 1 mM L-Glutamine, 50 μM β-ME, 50 ng/mL human macrophage colony-stimulating factor (M-CSF) (PeproTech, Rocky Hill, NJ, USA), and 25 ng/mL human IL-3 (Gibco) at 6–8 EB per well. The medium was changed every 3 days. Continuous monocyte production was cultured for 17–19 days. Non-adherent monocytes were collected, and the other cells were continuously cultured. Non-adherent monocytes were cultured in RPMI 1640 medium supplemented with 10% FBS, 100 ng/mL M-CSF, 50 ng/mL interleukin (IL)-3, and 50 μM β-ME and identified for Mφ after 10 days.

### THP-1 derived macrophages (THP-1- Mφ)

The THP-1 cells were trypsinized with 0.25% trypsin (Gibco) and centrifuged at 1000 rpm for 5 min. The cells were resuspended in RPMI 1640 medium supplemented with 100 ng/mL PMA (Beyotime, Beijing, China) and seeded into six-well plates at 10^6^ cells/well. After incubation for 24 h, the medium was discarded, the cells were washed once with phosphate-buffered saline (PBS), and fresh RPMI 1640 medium was added to the cells.

### Giemsa stain assay

iPS-Mφ and human monocyte cell line THP-1 (THP-1-Mφ) were fixed with 4% paraformaldehyde for 20 min. Subsequently, the cells were washed twice with PBS and incubated at room temperature (RT) for 10–15 min in Giemsa solution (Solarbio). The Giemsa solution was discarded, followed by washing in PBS. The results of cell staining were observed by an inverted microscope.

### Phagocytosis assay

iPS-Mφ and THP-1-Mφ were washed once in PBS. The Indian ink (Solarbio) was added to fresh medium at a ratio of 1:1000 and incubated with the cells for 1 h at 37 °C in a 5% CO_2_ incubator. The results of cell phagocytosis were observed and counted by an inverted microscope.

### Non-specific esterase stain (α-NAE method) assay

The cells were fixed for approximately 10–15 min in α-NAE solution (Solarbio). Then, the cells were washed in PBS. The cells were added to the configured α-NAE incubation solution, incubated for 1 h at 37 °C in the dark, and subsequently washed with PBS. The cells were stained for approximately 5–15 min by methyl green dyeing solution, washed, and recorded by microscopic examination.

### Flow cytometry

For the detection of surface markers and intracellular proteins, the cells were harvested after trypsin treatment and resuspended in ice-cold PBS. The following antibodies (Abs) conjugated with Annexin V-Fluorescein isothiocyanate (FITC) or PE were purchased from eBioscience (San Diego, CA, USA): anti-human CD11b FITC (11–0118), anti-human CD11b APC (17–0118), anti-human CD14 FITC (11–0149), anti-human CD40 PE (12–0401), anti-human CD68 PE (12–0689), and anti-mouse major histocompatibility complex (MHC) Class II (I-A) PE (12–5322). The cells were stained with the fluorochrome-conjugated Abs for 15 min and then washed twice with PBS containing 2% FCS. The stained cells were recorded using a FACS Calibur platform (BD Biosciences, San Jose, CA, USA) and analyzed using FlowJo software (Tree Star, Ashland, OR, USA).

### Immunostaining

For the detection of surface markers and intracellular proteins, the cells were washed twice with phosphate-buffered saline (PBS) and fixed in 4% paraformaldehyde in 0.1 M phosphate buffer (pH 7.4) for 10 min. The cells were washed three times with PBS, and blocked for at least 30 min with 1% BSA, 22.52 mg/ml glycine in PBST (PBS + 0.1% Tween 20) at room temperature. They were then incubated at 4 °C overnight with the following antibodies: rabbit anti-CD11b (1:250; Abcam, Cambridge, MA USA), rabbit anti-CD14(1:500; PeproTech), rabbit anti-CD68 (1:500; PeproTech), rabbit anti-MHC class II(1;250; Abcam). The cells were washed three times with PBS and then incubated with the following secondary antibodies: Alexa Fluor 555-labeled donkey anti-rabbit IgG (H + L) (1:500; Beyotime), nuclei were counterstained with 4′,6-diamidino-2-phenylindole (DAPI) (1 μg/ml; Beyotime). The plates were examined by inverted fluorescence microscopy (Olympus, Tokyo, Japan) and the images were processed using the Adobe Photoshop software (Adobe, San Jose, CA, USA). The antibody details are listed in Table 1.

**Table 1:** Primary antibodies used for immunostaining and FACS

Antibody	Company	Catalogue no.	Dilution
Anti-CD11b antibody	Abcam	ab52478	1:250
CD14 antibody	PeproTech	17,000–1-AP	1:500
Anti-CD40 antibody	Abcam	ab224639	1:100
CD68 antibody	PeproTech	25,747–1-AP	1:500
Anti-MHCII antibody	Abcam	ab157210	1:250
Alexa Fluor 555-labeled donkey anti-rabbit (H + L)	Beyotime	A0453	1:500
Mouse IgG1 kappa isotype control, APC	eBioscience	17–4714	1:20
Anti-human CD11b APC	eBioscience	17–0118	1:20
Anti-human CD14 APC	eBioscience	17–0149	1:20
Rat IgG2a K Iso control PE	eBioscience	12–4321	1:20
Anti-human CD40 PE	eBioscience	12–0401	1:40
Anti-human CD68 PE	eBioscience	12–0689	1:20
Anti-mouse MHC Class II (I-A) PE	eBioscience	12–5322	1:40

### BCG infection

iPS-Mφ and THP-1-Mφ (1 × 10^6^ cells/well) were infected with 1 × 10^7^ CFU of the BCG (http://www.shanghaishengwu.com). The culture systems were maintained according to the conditions described above. After 24 h, the supernatants and cells were collected separately for measurement. Untreated cells were used as controls.

### Apoptosis assay by flow cytometry

Apoptosis was performed by using a modified version of a previously established protocol [40]. Briefly, the cells were subsequently dissociated into single cells by trypsin, and apoptosis was measured according to the manufacturer’s instructions with the FITC Annexin V Apoptosis Detection Kit (BD Biosciences).The samples were detected by using flow cytometry.

### NO concentration detection

The supernatant of BCG-treated iPS-Mφ and THP-1-Mφ were collected separately, and the concentration of nitric oxide (NO) was determined by using the Griess Reagent System (Promega, Madison, WI, USA) according to the manufacturer’s instructions. Different concentrations of gradient nitrite standard solution were prepared. To generate the nitrite standard reference curve, the absorbance value of the nitrite standard solution was measured by a standard microplate reader at 540 nm. The absorbance value of the samples was measured, and the concentration was calculated according to the nitrite standard reference curve.

### Tumor necrosis factor alpha (TNF-α) assay

The supernatant of BCG-treated iPS-Mφ and THP-1-Mφ was collected, according to instructions of the mouse TNF-α ELISA Kit (R&D Systems, Minneapolis, MI, USA). The standard and sample wells were added to the corresponding wells of a microelisa strip plate, followed by the addition of HRP-labeled antibody. Chromogen Solution A and Chromogen Solution B were added to wells after washing and incubated in the dark. Stop solution was added, and the absorbance was measured with a standard microplate reader at a 450 nm wavelength. After drawing the standard linear regression curve of standards, the sample concentration was calculated according to the curve equation.

### Western blot analysis

Whole lysates of THP-1-Mφ and iPS-Mφ were extracted with a protein extraction kit according to the manufacturer’s instructions. Western blotting was performed following standard methods. Detailed antibody information is provided below: anti-Actin (Sigma-Aldrich, dilution ratio 1:1000), anti-TNF-α (Sigma-Aldrich, dilution ratio 1:1000), anti-apoptosis-related protein cysteine-3 (Caspase-3) (Sigma-Aldrich, dilution ratio 1:1000), and anti-B-cell lymphoma-2 (Bcl-2) (Sigma-Aldrich, dilution ratio 1:1000).

### Statistical analysis

All statistical analyses were performed with SPSS version 13.0 software (SPSS, Inc., Chicago, IL, USA). Two-tailed unpaired Student’s *t* tests were used to evaluate differences between the control and treated groups. The data were given as the means of three experiments with *n* = 3. The data are presented as the means ± standard deviation. A value of *P* < 0.05 represents a statistical difference, and *P* < 0.01 represents a statistically significant difference.

## Results

### Differentiation of hiPS into Mφ

**Fig. 1:**
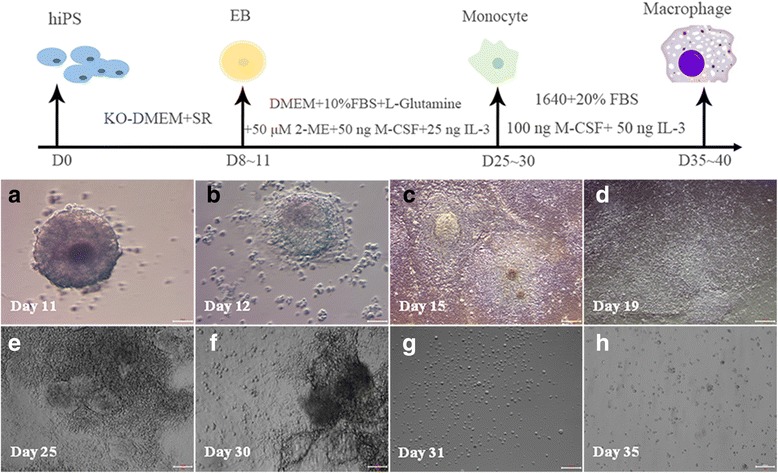
Differentiation of iPS into Mφ. The *upper panel* is a schematic representation of the three-step EB-based protocol employed for hematopoietic differentiation. **a** Embryoid bodies (EB) are formed for 11 days; (**b**) EB started to gradually adhere in the presence of 25 ng/ml IL-3 with 50 ng/ml M-CSF added from day 12 onward; (**c**) Most EB were propagated in the presence of 25 ng/ml IL-3 with the addition of 50 ng/ml M-CSF from day 15 onward; (**d**) all EB were propagated and adherent in the presence of 25 ng/ml IL-3 with the addition of 50 ng/ml M-CSF from day 19 onward; (**e**-**f**) The cells were cultured for 25–30 days and were differentiated into monocytes (some round suspension cells were present), and suspension cells were collected every 4–5 days and changed to fresh medium; (**g**) collected suspension cells were cultured in the presence of 50 ng/ml IL-3 with 100 ng/ml M-CSF; (**h**) terminal differentiation is achieved by 35–40 days of adherent culture in the presence of 50 ng/ml IL-3 with 100 ng/ml M-CSF. The cells have typical macrophage morphological characteristics (round, oval, spindle and irregular, etc., and some cells showed pseudopods and convex forms)

Differentiation studies of hiPS into Mφ have utilized an EB-based protocol. EB in suspension cultures were incubated in an orbital shaker, and to achieve terminal Mφ differentiation in secondary cultures, M-CSF and interleukin-3 (mIL-3) treatment was employed from days approximately 8–11 onward. The cells grew rapidly when EB were adherent cultured for approximately 15 days as shown in Fig. 1a-c). After approximately 8–11 days, the medium was replaced with monocyte differentiation medium employing M-CSF and interleukin-3 (mIL-3), and the cells showed irregular differentiation as shown in Fig. 1d. At 25–30 days, the cells tended towards monocyte differentiation, and the culture system showed round, oval cells (as shown in Fig. 1e-f). The suspension cells were collected for further differentiation as shown in Fig. 1g. When employing a higher concentration (twofold) of M-CSF and IL-3 in the cultures, the round and oval-shaped cells gradually increased at approximately day 35, at which time the cells displayed plastic adherence and Mφ-typical morphology (> 95% purity), displaying round, oval, spindle-shaped and irregularly shaped cells. Some cells were observed as convex pseudopods with other typical Mφ morphological characteristics at approximately day 35–40 upon bright-field microscopy, as shown in Fig. 1h.

### Functional characterization of hiPS-Mφ

**Fig. 2:**
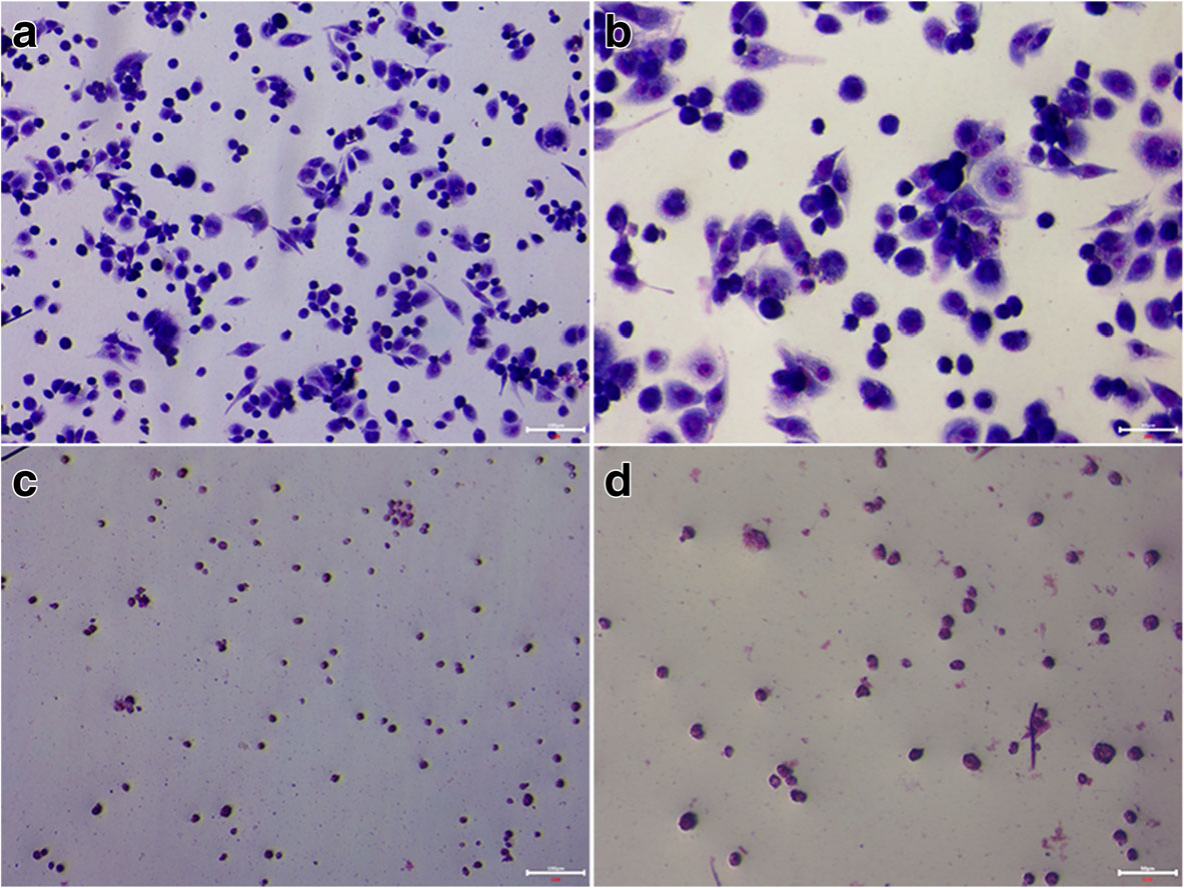
Giemsa stain. **a** THP-1-Mφ were positive by Giemsa stain. Bar = 100 μm; (**b**) THP-1-Mφ were positive by Giemsa stain. Bar = 50 μm; (**c**) iPS-Mφ were positive by Giemsa stain. Bar = 100 μm. **d** iPS-Mφ were positive by Giemsa stain. Bar = 50 μm

**Fig. 3:**
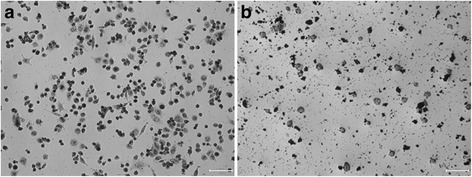
α-NAE stain. **a** THP-1-Mφ were positive by α-NAE stain. Bar = 50 μm; (**b**) iPS-Mφ were positive by α-NAE stain. Bar = 50 μm

The Mφ characteristics of almost all cells (> 95% purity) were verified by typical, spread-out morphology with bright-field microscopy, and strong adherence to non-tissue culture-treated plates as well as a classical Mφ phenotype on Giemsa-stained cytospins resembling the THP-1-Mφ used as controls. The stained cells had clear circular or irregular shapes, with pseudopods and protrusions. The cells had rich cytoplasm and a larger nucleus. The nucleus was located at one end of the cell and appeared darker. The morphology of the cells was horseshoe, round or irregular-shaped, as shown in Fig. 2. Additionally, iPS-Mφ and THP-1-Mφ were collected for identification by non-specific esterase stain. As shown in Fig. 3, after the cells were stained with non-specific esterase, we observed black or brown-black dispersible particles in the cytoplasm, indicating that the cells contained α-acetate esterase.

**Fig. 4:**
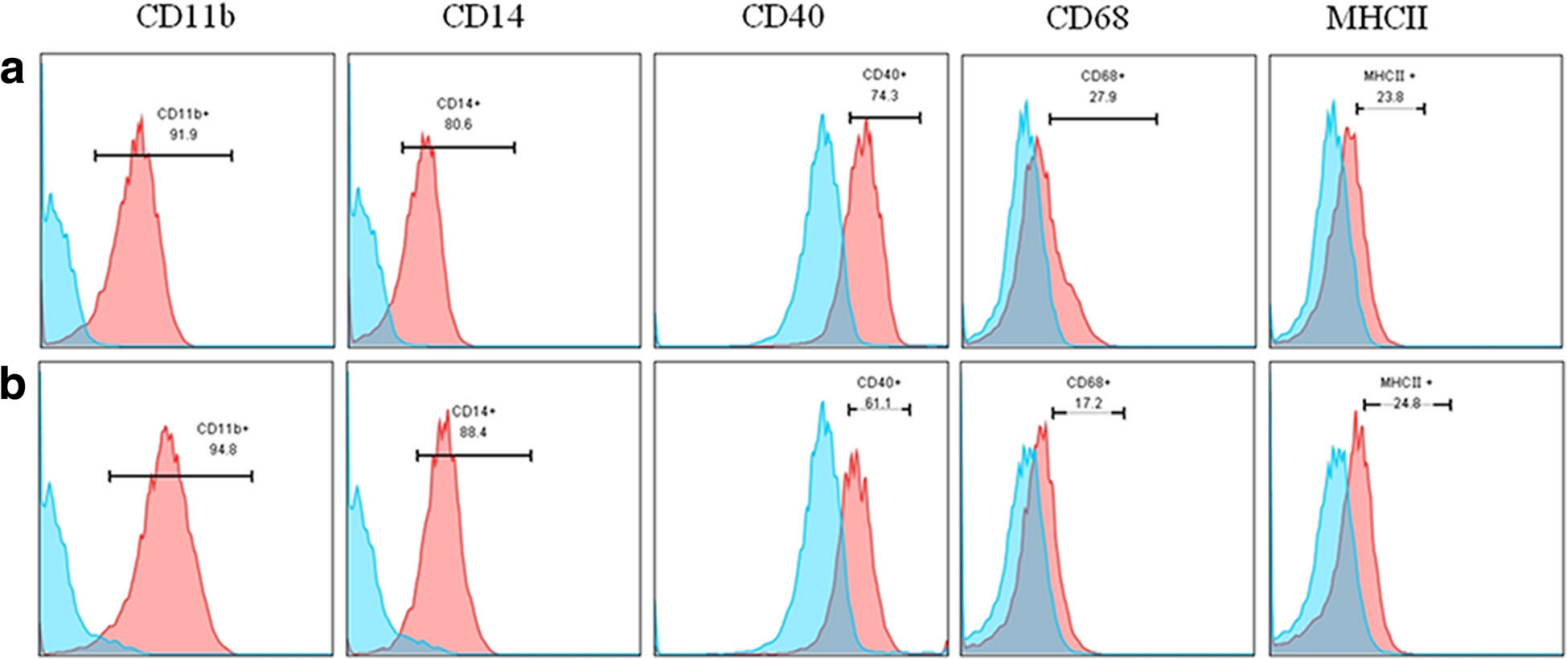
Flow cytometry analysis of surface phenotypes and major histocompatibility complex (MHC-II) expression of iPS-Mφ, THP-1-Mφ were used as control. The *upper panel* (**a**) shows that iPS-Mφ expressed macrophage-specific markers CD11b, CD14, CD40, CD68, and major histocompatibility complex (MHC-II); the *lower panel* (**b**) shows that THP-1-Mφ expressed the macrophage-specific markers CD11b, CD14, CD40, CD68, and major histocompatibility complex (MHC-II)

According to the antibody properties of the labeled anti-macrophage surface-specific antigen, the percentage of positive cells expressing CD11b, CD14, CD40, CD68, and MHC-II proteins were detected by flow cytometry at the corresponding wave lengths. The results showed that iPSC-Mφ displayed the classical Mφ marker. The percentage of cells expressing CD11b, CD14, CD40, CD68, and MHC-II surface antigens was 91.9%, 80.6%, 74.3%, 27.9%, and 23.8% in iPS-Mφ, respectively. By comparison, THP-1-Mφ antigen expression was similar to iPS-Mφ, as shown in Fig. 4. Furthermore, immunostaining results showed that (Additional file 1: Figure S1, Additional file 2: Figure S2, Additional file 3: Figure S3, Additional file 4: Figure S4 and Additional file 5: Figure S5), the expression of Mφ lineage markers CD11b, CD14, CD40, CD68, and MHC-II were all similar in iPSC-Mφ and in THP-1-Mφ as well as in hES-derived macrophages (ES-Mφ). These results demonstrated that the induced and differentiated cells are macrophages.

**Fig. 5:**
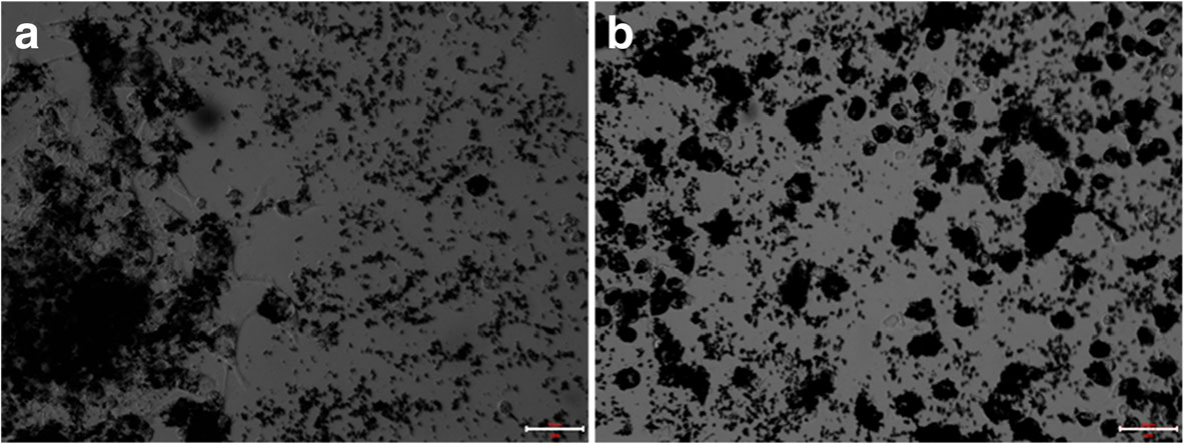
Phagocytic function test. **a** iPS-Mφ, Bar = 50 μm. **b** THP-1-Mφ, Bar = 50 μm

The phagocytosis function of iPS-Mφ was tested by the phagocytic ability of ink particles, and THP-1-Mφ was used as a control. As shown in Fig. 5, a varying degree of ink particles was distributed throughout the cytoplasm, indicating that the cells exhibited phagocytic ability.

### Immunological function of iPS-Mφ in response to tuberculosis infection

**Fig. 6:**
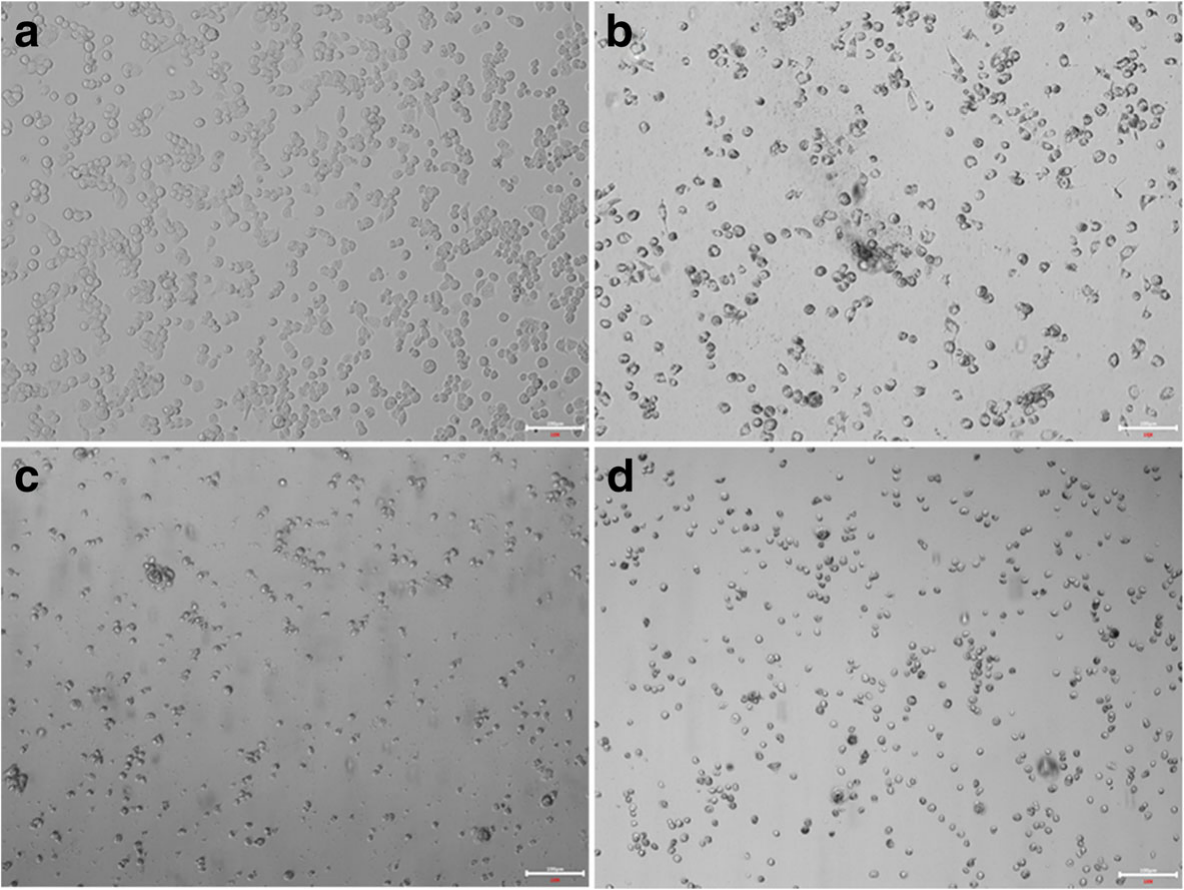
Morphological changes of iPS-Mφ and THP-1-Mφ after treatment with BCG for 24 h. **a** Untreated THP-1-Mφ, Bar = 100 μm; (**b**) THP-1-Mφ treated with BCG, Bar = 100 μm; (**c**) untreated iPS-Mφ, Bar = 100 μm; (**d**) iPS-Mφ treated with BCG, and iPS-Mφ were shrunken after BCG treatment for 24 h. Bar = 100 μm

To determine whether iPS-Mφ would show the same response to tuberculosis infection as THP-1- Mφ, we treated iPS-Mφ with BCG for 24 h and then observed the morphology of iPS-Mφ. The morphology of iPS-Mφ was changed after BCG treatment for 24 h. As shown in Fig. 6, iPS-Mφ were shrunken, and some of the cells were ruptured, similar to THP-1-Mφ.

**Fig. 7:**
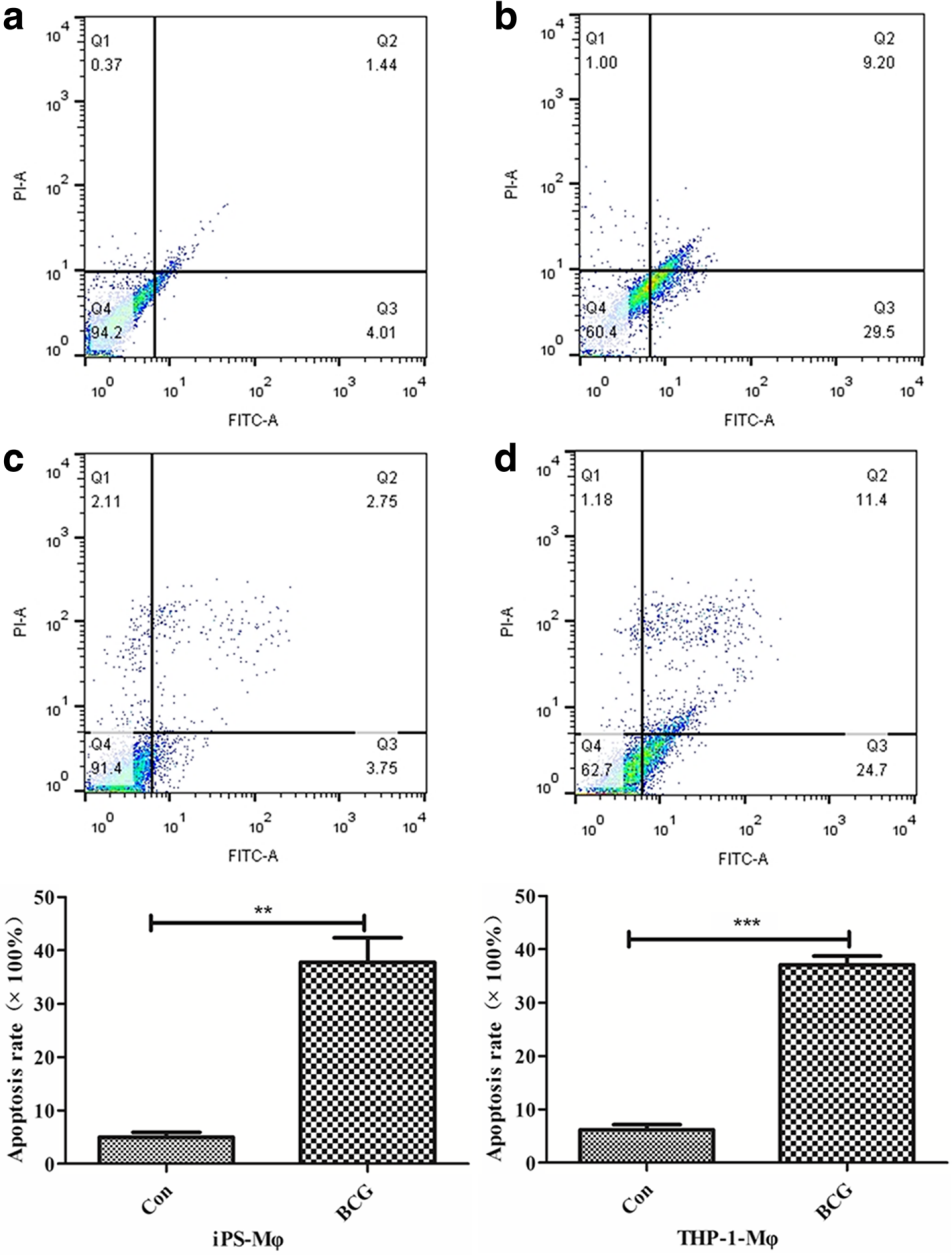
BCG induced iPS-Mφ apoptosis by flow cytometric analysis of FITC Annexin V staining. iPS-Mφ were treated with BCG for 24 h. FITC Annexin V and propidium iodide (PI)-positive cells were analyzed by flow cytometry. The majority (37.77 ± 7.94%, *n* = 3) of treated iPS-Mφ became PI positive, FITC Annexin V positive, or both FITC Annexin V and PI positive. In contrast, only a small portion (4.97 ± 1.60%) of untreated iPS-Mφ was PI positive, FITC Annexin V positive, or both FITC Annexin V and PI positive (*P* < 0.01). The portion of treated THP-1-Mφ was 37.1 ± 2.84% and untreated THP-1-Mφ was 6.19 ± 1.68%. Statistical analysis was performed by using the *t* test. A value of *P* < 0.01 represents a statistically significant difference (*Upper panel*: upper right quadrant refers to late apoptosis; upper left quadrant refers to necrotic cells; lower left quadrant refers to viable cells; lower right quadrant refers to early apoptosis). *TUNEL* terminal deoxynucleotidyl transferase dUTP nick end labeling. The apoptosis analysis was repeated at least three times and demonstrated consistent results. Note: the upper panel: **a** was untreated iPS-Mφ, **b** was treated iPS-Mφ, **c** was untreated THP-1-Mφ, **d** was treated THP-1 -Mφ; the lower panel was the ratio of the apoptosis

To further determine how iPS-Mφ responded to tuberculosis challenge, iPS-Mφ were treated with BCG for 24 h, and the apoptosis rates were evaluated by using a FITC Annexin V Apoptosis Detection Kit. According to manufacturer instructions, the cells in late apoptosis or already dead cells are both FITC Annexin V and propidium iodide (PI) positive, whereas cells undergoing apoptosis are FITC Annexin V positive. iPS-Mφ and THP-1- Mφ were incubated with FITC Annexin V in a buffer containing PI and subsequently analyzed by flow cytometry. The apoptosis analysis was repeated at least three times and demonstrated consistent results. As shown in Fig. 7, the majority (37.77 ± 7.94%, *n* = 3) of treated iPS-Mφ became PI positive, FITC Annexin V positive, or both FITC Annexin V and PI positive. In contrast, only a small portion (4.97 ± 1.60%) of untreated iPS-Mφ was PI positive, FITC Annexin V positive, or both FITC Annexin V and PI positive (*P* < 0.01). Notably, the portion of treated THP-1-Mφ was 37.1 ± 2.84% and the portion of untreated THP-1-Mφ was 6.19 ± 1.68%. Thus, iPS-Mφ performed similar to THP-1-Mφ in response to BCG.

**Fig. 8:**
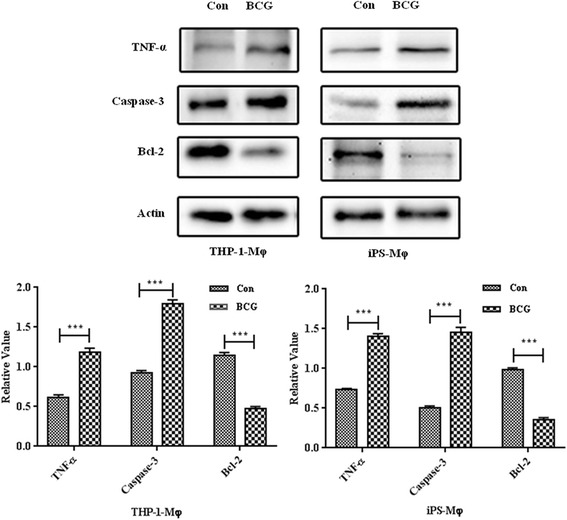
Western blot analysis of TNF-α, Caspase 3, and Bcl-2 expression in iPS-Mφ and THP-1-Mφ treated with BCG (BCG) for 24 h, untreated iPS-Mφ and THP-1-Mφ were used as controls (Con; *upper panel*). TNF-α, Caspase 3, and Bcl-2 were analyzed in whole-cell protein extracts. Actin was used as the loading control. All expression values were normalized against actin as the endogenous control. The results were the average of three independent experiments.***P < 0.001. A value of *P* < 0.001 is set to represent a statistically significant difference

To investigate how apoptosis is initiated and determine the cytokine involved profile, we measured the protein expression levels of Bcl-2, Caspase-3, and TNF-α after iPS-Mφ were cultured in the presence of BCG for 24 h. As shown in Fig. 8, the expression levels of TNF-α and Caspase-3 in iPS-Mφ and THP-1-Mφ were increased after BCG treatment compared with the untreated group; however, the level of Bcl-2 was decreased (*P* < 0.001).

**Fig. 9:**
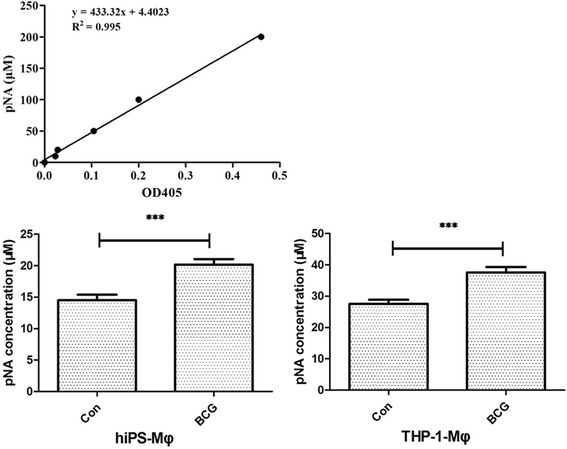
BCG treatment increased the Caspase-3 activity of iPS-Mφ. iPS-Mφ and THP-1-Mφ were treated by BCG for 24 h. The *upper panel* illustrates the determination of the standard curve of Caspase-3, whereas the *lower panel* documents Caspase-3 activity. The majority (20.17 ± 0.8696 μM) of BCG-treated iPS-Mφ (BCG) became p-nitroaniline (pNA) positive. In contrast, only a small portion (14.52 ± 0.8696 uM) of iPS-Mφ untreated with BCG (Con) was pNA positive (*P* < 0.001).The majority (37.57 ± 1.739 uM) of BCG-treated THP-1-Mφ (BCG) became pNA positive. In contrast, only a small portion (27.57 ± 1.304 uM) of iPS-Mφ untreated with BCG (Con) was pNA positive (*P* < 0.001).Statistical analysis was performed by using a *t* test. A value of *P* < 0.001 represents a statistically significant difference

Because Caspase-3 is a central caspase in the pro-apoptotic cascade and plays a key role in various forms of apoptosis, the Caspase-3 activity of iPS-Mφ and THP-1-Mφ was therefore determined by the Caspase-3 Activity Assay Kit. The results showed that the BCG treatment increased Caspase-3 activity in iPS-Mφ, similar to THP-1-Mφ as shown in Fig. 9.

**Fig. 10:**
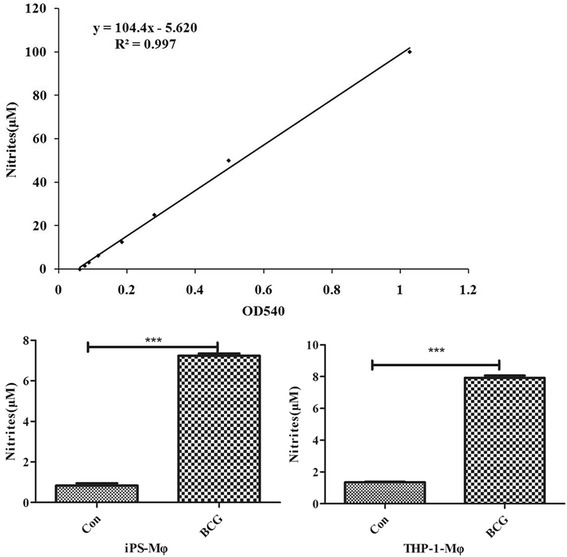
Effects of BCG on the production of NO in iPS-Mφ and THP-1-Mφ. The *upper panel* was the standard curve of NO. The production of NO in BCG-treated iPS-Mφ and THP-1-Mφ was significantly increased (*P* < 0.001)

We further examined the cytokine profiles in both iPS-Mφ and THP-1 cells after infection with BCG for 24 h. The content of NO in different concentration standards was measured by using the Griess method. The results are shown in Fig. 10. Compared with the control group, the production of NO in BCG-treated iPS-Mφ and THP-1-Mφ was significantly increased (*P* < 0.001).

**Fig. 11:**
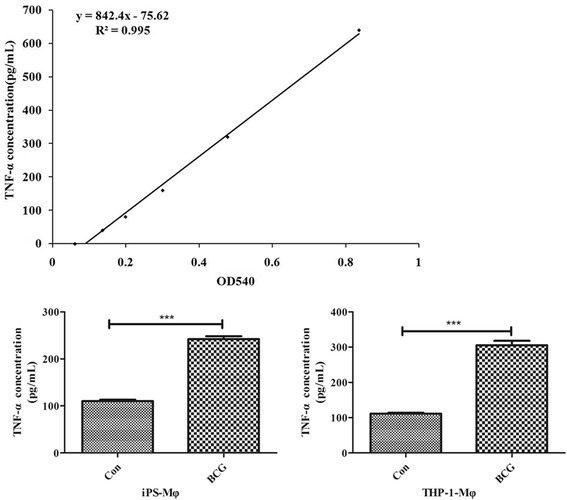
Effects of BCG on TNF-α expression in iPS-Mφ and THP-1-Mφ. The *upper panel* shows the standard curve of TNF-α. The expression of TNF-α protein in iPS-Mφ and THP-1-Mφ was significantly increased after BCG treatment (*P* < 0.001)

The production of TNF-α was measured by using an ELISA Kit. As shown in Fig. 11, the expression of TNF-α protein in iPS-Mφ and THP-1-Mφ was significantly increased after BCG treatment (*P* < 0.001).

## Discussion

**Fig. 12:**
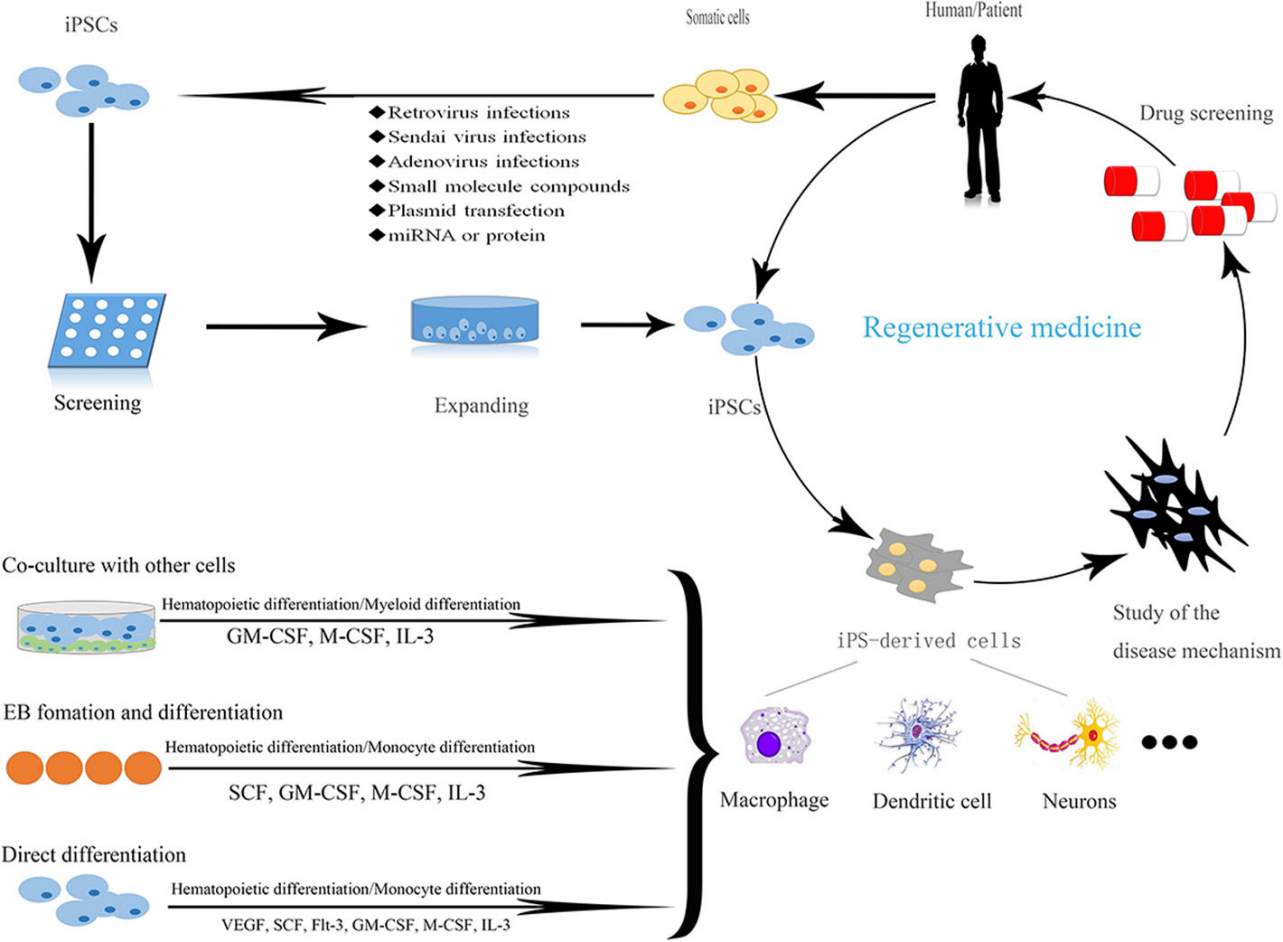
Potential application of iPS in regenerative medicine and differentiation into macrophages

In an ideal world, nearly any cell type necessary for the analysis of host-pathogen interactions or therapy *in vitro* could be generated from stem cells [41]. Among most stem cell types, hiPS-based gene and cell therapy holds great promise for innovative treatment strategies in regenerative medicine. The concepts most commonly envisioned in this context postulate the generation of patient-derived iPS, which, after appropriate differentiation, will be transplanted to the patient as individual-specific treatments or for the screening of drugs (as shown in Fig. 12) [42]. The efficient differentiation of iPS has previously been established for a number of different tissues and organ systems, including the cells of the hematopoietic system [17]. However, iPS-based gene and cell therapy approaches employing patient-specific iPS rely on the efficient robustness and reproducibility of the differentiation of target cells.

Among the multiple lineages of differentiated cells derived from hiPS, macrophages hold great promise in disease pathogenesis or therapy, such as cancer [29, 30], Mendelian disease [31], Alzheimer’s disease [30], HIV [32], *Chlamydia trachomatis* pathogenesis [33], chronic granulomatous disease [34], and X-linked chronic granulomatous diseases [35], particularly tuberculosis. Despite substantial appreciation for the dual function of macrophages in innate immunity and lipid metabolism, understanding human macrophage biology has been hampered by the lack of reliable and scalable models for cellular and genetic studies. In the present study, we generated macrophages derived from hiPS. These iPS-derived macrophages exhibited the typical morphology of macrophages and were tested for the expression of cell surface markers (e.g., CD11b, CD14, CD40, CD68) and major histocompatibility complex (MHC-II) and phagocytosis of foreign particles. The iPS-derived macrophages were not distinguishable from THP-1-derived macrophages. We also figured out that based on the same differentiation method, human ES could differentiate into macrophage. Moreover, human ES derived-macrophages expressed cell surface markers (CD11b, CD14, CD40, CD68) and major histocompatibility complex (MHC-II), which were all similar to iPSC-Mφ.

Tuberculosis is a zoonotic infectious disease that poses a serious threat to human health. As the main host cells to invasive MTB, macrophages interact with MTB and, therefore, play a crucial role in the occurrence and development of tuberculosis. Studies of their interactions have confirmed that these cells indeed play a crucial role in the occurrence and development of tuberculosis.

Bacteria and other pathogens are recognized via their pathogen-associated molecular patterns, which initiate a signaling cascade that results in phagocytosis of pathogens and upregulation of a pro-inflammatory response. Macrophages kill the infected MTB by direct phagocytosis, autophagy, apoptosis, and secretion of inflammatory factors and free radicals. MTB infection results in the activation of macrophages that secrete a series of inflammatory factors, such as TNF-α, IL-6, etc. TNF-α is an important inflammatory factor that regulates cellular non-specific immunity to MTB and cell apoptosis. Activated macrophages kill MTB by catalyzing NO. Moreover, MTB regulate cell apoptosis mainly via the Caspase family, Bcl family, TNF-α, NO, and other pathways. Therefore, to further elucidate the immunological function of hiPS-derived macrophages in response to tuberculosis infection, we examined the effects of BCG on iPS-Mφ. Our results demonstrate that in response to BCG infection, iPS-Mφ undergo apoptosis and increase the production of NO and expression of TNF-α. Additionally, our results demonstrate that apoptosis was induced by the suppressed expression of Bcl-2 and enhanced activity of Caspase-3. The iPS-derived macrophages were not distinguishable from THP-1-derived macrophages in response to tuberculosis infection. The most notable cytokines produced by macrophages associated with this pro-inflammatory state in TB are tumor necrosis factor (TNF) [43]. Our results are consistent with those of a previous study [43]. Therefore, we have provided evidence for the use of human iPS-derived macrophages to study the complex interplay between the host and pathogen after tuberculosis infection.

## Conclusions

In conclusion, human iPS-derived macrophages respond to BCG in a similar manner to macrophages derived from human monocytes. Importantly, the identification host factors potentially involved in macrophage–mycobacterium tuberculosis interaction. Thus, we have provided evidence that we can use the human iPS-Mφ in response to the Bacillus Calmette-Guérin infection model to study the complex interplay between the host and the pathogen.

## Additional files

Additional file 1: **Figure S1.** Immunofluorescence images showing the positive expression of Mφ lineage markers CD11b in iPS-Mφ (A), THP-1-Mφ (B) and ES-Mφ (C). Nuclei are labeled with DAPI. Bar = 100 μm. (TIFF 1395 kb)

Additional file 2: **Figure S2.** Immunofluorescence images showing the positive expression of Mφ lineage markers CD14 in iPS-Mφ (A), THP-1-Mφ (B) and ES-Mφ (C). Nuclei are labeled with DAPI. Bar = 100 μm. (TIFF 1337 kb)

Additional file 3: **Figure S3.** Immunofluorescence images showing the positive expression of Mφ lineage markers CD40 in iPS-Mφ (A), THP-1-Mφ (B) and ES-Mφ (C). Nuclei are labeled with DAPI. Bar = 100 μm. (TIFF 1548 kb)

Additional file 4: **Figure S4.** Immunofluorescence images showing the positive expression of Mφ lineage markers CD68 in iPS-Mφ (A), THP-1-Mφ (B) and ES-Mφ (C). Nuclei are labeled with DAPI. Bar = 100 μm. (TIFF 1431 kb)

Additional file 5: **Figure S5.** Immunofluorescence images showing the positive expression of Mφ lineage markers MHC-II in iPS-Mφ (A), THP-1-Mφ (B) and ES-Mφ (C). Nuclei are labeled with DAPI. Bar = 100 μm. (TIFF 1462 kb)

## Abbreviations

Abs: Antibodies
BCG: Bacillus Calmette-Guérin
Bcl-2: B-cell lymphoma-2
Caspase-3: Apoptosis-related protein cysteine-3
DAPI: 4′,6-diamidino-2-phenylindole
EB: Embryoid body
ES-Mφ: hES-derived macrophages
FBS: Fetal bovine serum
FITC: Annexin V-Fluorescein isothiocyanate
hES: Human embryonic stem cells
IL: Interleukin
iPS: Induced pluripotent stem cells
iPS-Mφ: hiPS-derived macrophages
M-CSF: Macrophage colony-stimulating factor
MHC-II: Major histocompatibility complex II
MTB: Mycobacterium tuberculosis
Mφ: Macrophages
NO: Nitric oxide
OP9: Bone marrow stromal cells
PBS: Phosphate-buffered saline
PI: Propidium iodide
pNA: p-nitroaniline
THP-1-Mφ: Human monocyte cell line THP-1
TNF-α: Tumor necrosis factor alpha
α-NAE: Nonspecific esterase staining
