# Supplementary figures and images for: Human-induced pluripotent stem cell-derived macrophages and their immunological function in response to tuberculosis infection
Source: Stem Cell Res Ther. 2018 Feb 26;9:49. doi: 10.1186/s13287-018-0800-x (PMC5828072; doi:10.1186/s13287-018-0800-x)

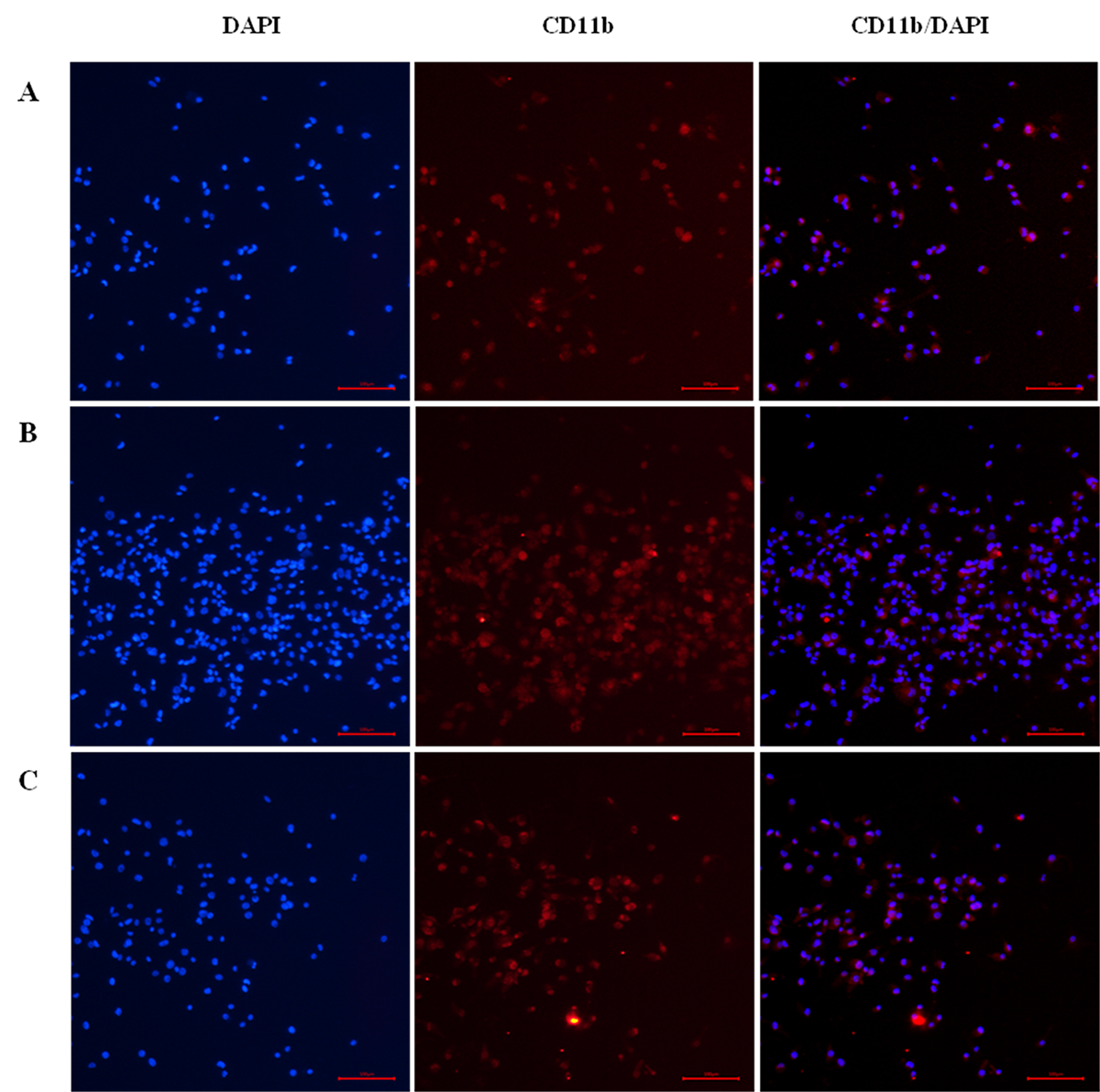

Supplement: Supplementary file 1 — Figure S1. Immunofluorescence images showing the positive expression of Mφ lineage markers CD11b in iPS-Mφ (A), THP-1-Mφ (B) and ES-Mφ (C). Nuclei are labeled with DAPI. Bar = 100 μm. (TIFF 1395 kb) [file 13287_2018_800_MOESM1_ESM.tif]

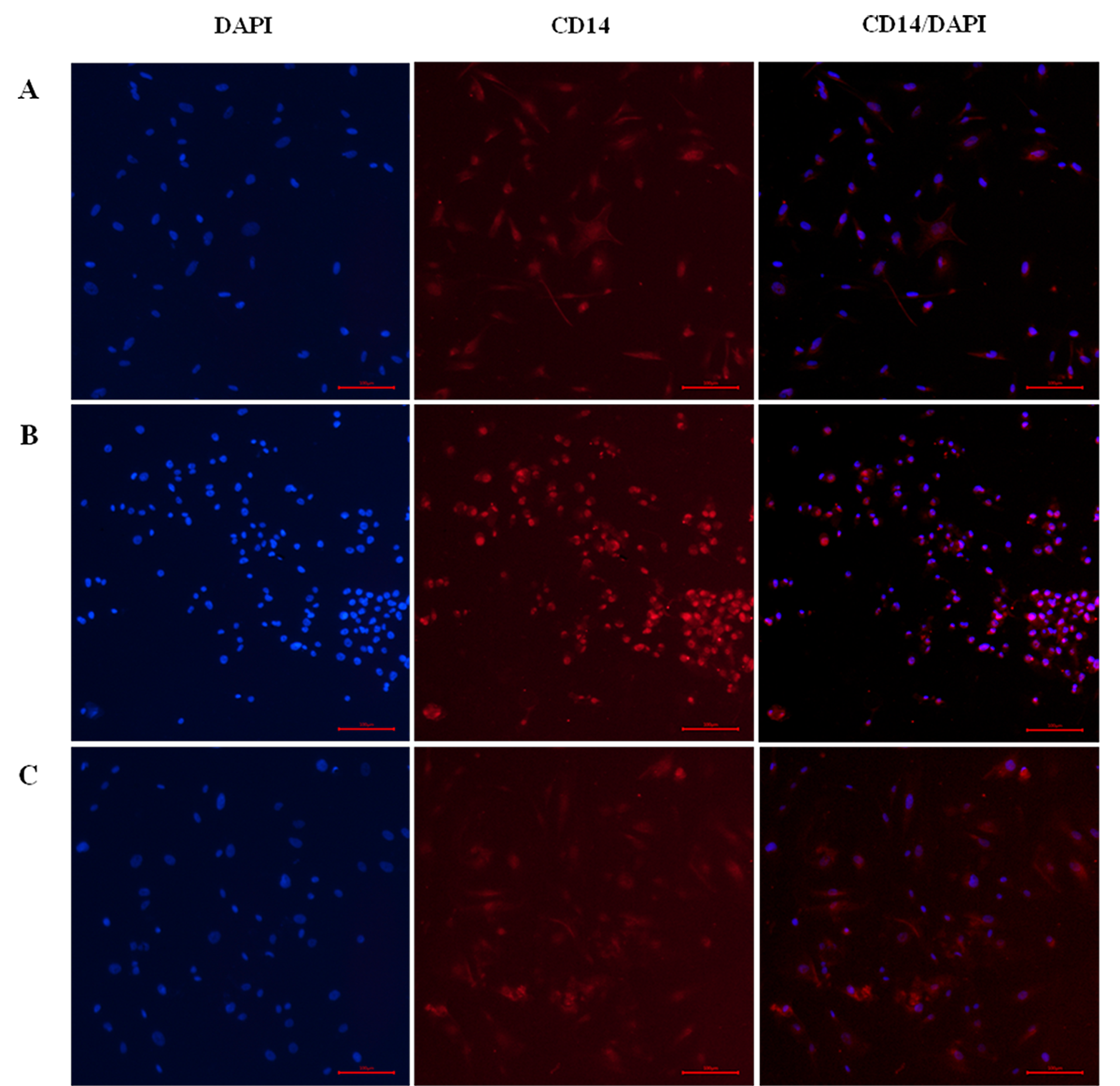

Supplement: Supplementary file 2 — Figure S2. Immunofluorescence images showing the positive expression of Mφ lineage markers CD14 in iPS-Mφ (A), THP-1-Mφ (B) and ES-Mφ (C). Nuclei are labeled with DAPI. Bar = 100 μm. (TIFF 1337 kb) [file 13287_2018_800_MOESM2_ESM.tif]

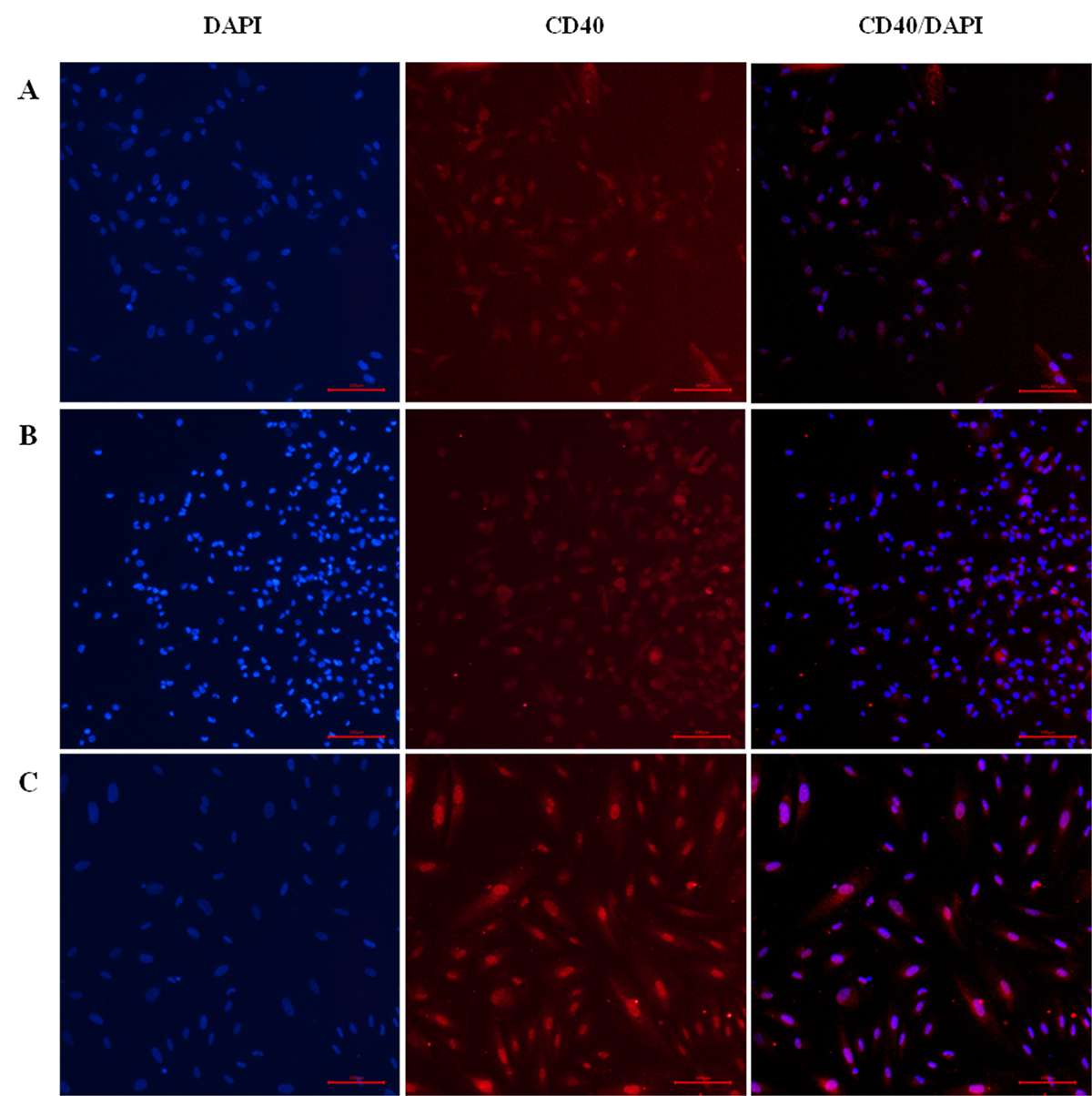

Supplement: Supplementary file 3 — Figure S3. Immunofluorescence images showing the positive expression of Mφ lineage markers CD40 in iPS-Mφ (A), THP-1-Mφ (B) and ES-Mφ (C). Nuclei are labeled with DAPI. Bar = 100 μm. (TIFF 1548 kb) [file 13287_2018_800_MOESM3_ESM.tif]

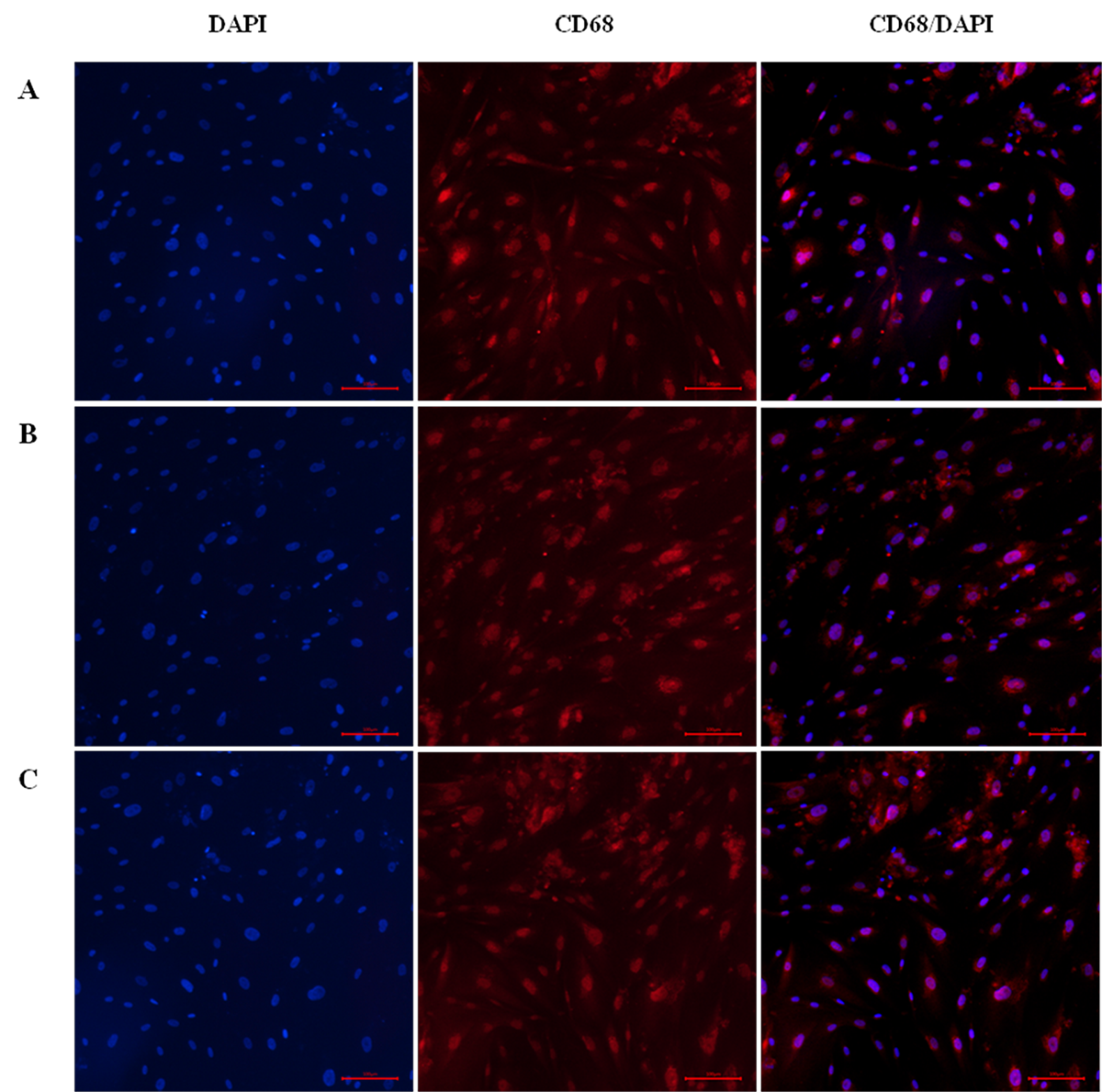

Supplement: Supplementary file 4 — Figure S4. Immunofluorescence images showing the positive expression of Mφ lineage markers CD68 in iPS-Mφ (A), THP-1-Mφ (B) and ES-Mφ (C). Nuclei are labeled with DAPI. Bar = 100 μm. (TIFF 1431 kb) [file 13287_2018_800_MOESM4_ESM.tif]

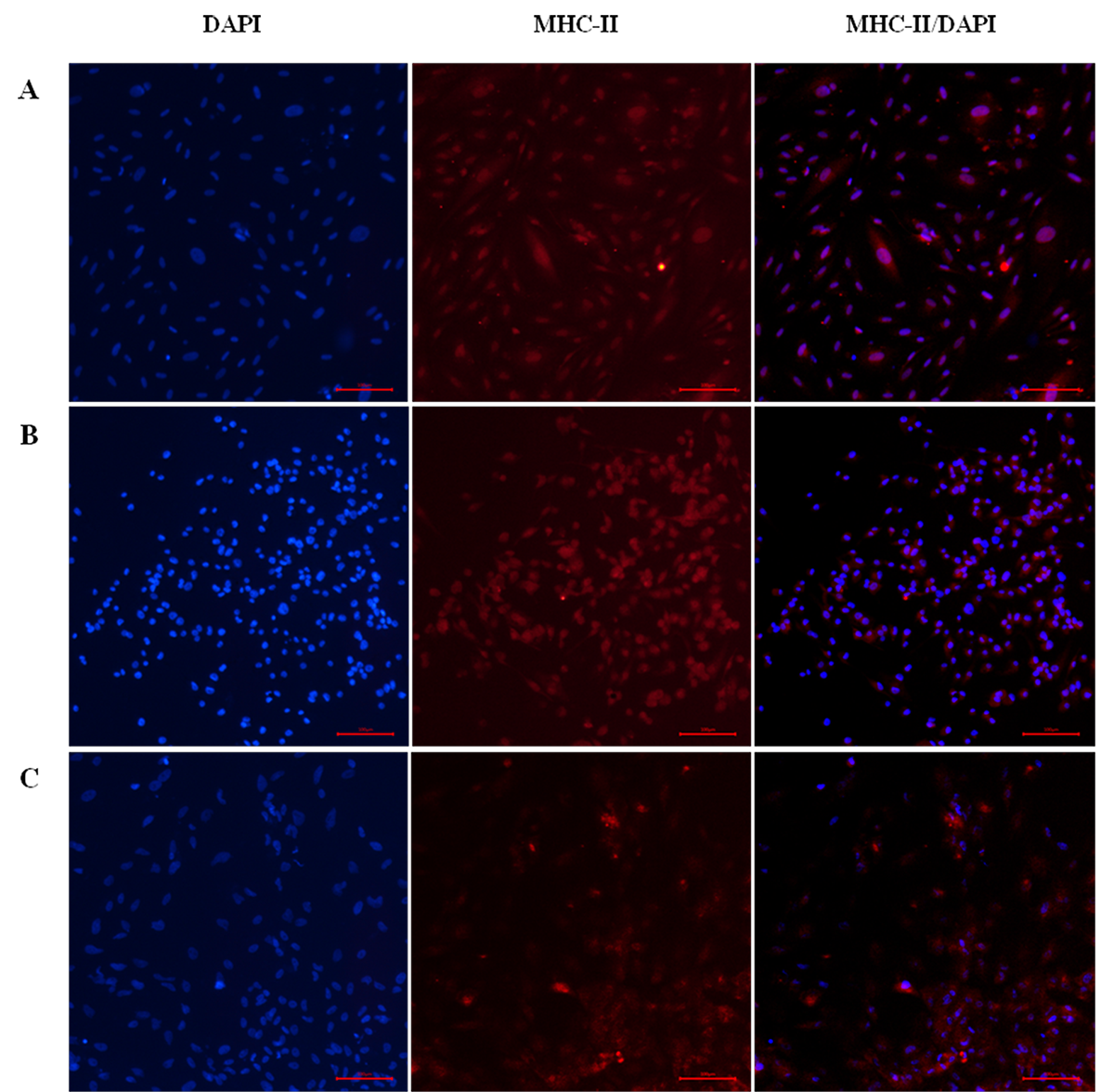

Supplement: Supplementary file 5 — Figure S5. Immunofluorescence images showing the positive expression of Mφ lineage markers MHC-II in iPS-Mφ (A), THP-1-Mφ (B) and ES-Mφ (C). Nuclei are labeled with DAPI. Bar = 100 μm. (TIFF 1462 kb) [file 13287_2018_800_MOESM5_ESM.tif]
